# Effects of an Additional Sequence of Color Stimuli on Visuomotor Sequence Learning

**DOI:** 10.3389/fpsyg.2017.00937

**Published:** 2017-06-13

**Authors:** Kanji Tanaka, Katsumi Watanabe

**Affiliations:** ^1^Faculty of Science and Engineering, Waseda UniversityTokyo, Japan; ^2^Research Center for Advanced Science and Technology, The University of TokyoTokyo, Japan; ^3^Japan Society for the Promotion of ScienceTokyo, Japan

**Keywords:** sequential learning, color cue, speed, accuracy, trial-and-error

## Abstract

Through practice, people are able to integrate a secondary sequence (e.g., a stimulus-based sequence) into a primary sequence (e.g., a response-based sequence), but it is still controversial whether the integrated sequences lead to better learning than only the primary sequence. In the present study, we aimed to investigate the effects of a sequence that integrated space and color sequences on early and late learning phases (corresponding to effector-independent and effector-dependent learning, respectively) and how the effects differed in the integrated and primary sequences in each learning phase. In the task, the participants were required to learn a sequence of button presses using trial-and-error and to perform the sequence successfully for 20 trials (*m* × *n* task). First, in the baseline task, all participants learned a non-colored sequence, in which the response button always turned red. Then, in the learning task, the participants were assigned to two groups: a colored sequence group (i.e., space and color) or a non-colored sequence group (i.e., space). In the colored sequence, the response button turned a pre-determined color and the participants were instructed to attend to the sequences of both location and color as much as they could. The results showed that the participants who performed the colored sequence acquired the correct button presses of the sequence earlier, but showed a slower mean performance time than those who performed the non-colored sequence. Moreover, the slower performance time in the colored sequence group remained in a subsequent transfer task in which the spatial configurations of the buttons were vertically mirrored from the learning task. These results indicated that if participants explicitly attended to both the spatial response sequence and color stimulus sequence at the same time, they could develop their spatial representations of the sequence earlier (i.e., early development of the effector-independent learning), but might not be able to enhance their motor representations of the sequence (i.e., late development of the effector-dependent learning). Thus, the undeveloped effector-dependent representations in the colored sequence group directly led to a long performance time in the transfer sequence.

## Introduction

Learning behavioral sequences, such as typing on a keyboard, is important in our daily life. Studies have investigated how people implicitly or explicitly learn a sequence by adopting various experimental paradigms. These paradigms include, for example, artificial grammar learning (Reber, 1967), the discrete sequence production task (Verwey, 1999), the visuomotor button press task (hereafter called the *m* × *n* task; Hikosaka et al., 1995), and the serial reaction time (SRT) task (Nissen and Bullemer, 1987). For example, in a typical SRT task, visual stimuli are successively presented at one of four or six horizontally aligned locations, and participants respond with spatially compatible key presses as quickly and accurately as possible (Nissen and Bullemer, 1987). Although they are not aware that a pre-determined sequence, typically composed of 8–12 key presses, was repeated during the experiment, their reaction times gradually become shorter, and are shorter than for randomly presented sequences. This reflects implicit learning of the sequence. As such, previous studies have investigated whether implicit sequence learning in the SRT task is mainly stimulus- or response-based learning (for overviews, see Abrahamse et al., 2010). To this end, Gheysen et al. (2009) devised a serial color-matching task and found that participants implicitly learned the stimulus-based sequence of colors without any specific responses, and learned the response-based sequence without any specific colored sequences. These results indicate that participants are able to learn both stimulus- and response-based sequences, although they are not aware of the sequence rule.

Taking advantage of both stimulus- and response-based learning, several studies have examined the effects of a secondary sequence on a primary sequence (e.g., Cock and Meier, 2013). For example, in Cock and Meier (2013), two types of visual stimuli (red or blue asterisks) were simultaneously presented at two out of four possible locations. Participants were instructed to attend to a specific color asterisk and to ignore the other one. In the correlated sequence condition, the attended sequence (response-relevant sequence) and ignored sequence (response-irrelevant sequence) were of the same length (e.g., 123243 for the response-relevant sequence and 241321 for the response-irrelevant sequence). In this example, when a participant presses the “2” location in the response-relevant sequence, a stimulus in the response-irrelevant sequence is shown at the “4” or “3” location. That is, the response-relevant and response-irrelevant sequences were predictable, but did not have a one-to-one association. In contrast, in the uncorrelated sequence condition, the sequences were of different lengths (e.g., six elements vs. seven elements; no predictable association). They found that, in the correlated condition, performance times were disrupted when the response-irrelevant sequence became a randomly generated sequence, while the response-relevant sequence was not changed. In contrast, in the uncorrelated condition, performance times were not disrupted. This result indicates that only when the response-relevant and irrelevant sequences share a predictable association (e.g., the same length of the sequence), the response-irrelevant sequence is integrated into the response-relevant sequence through intensive practice. According to the dual system model of sequence learning proposed by Keele et al. (2003), a set of unidimensional modules detect and utilize all available regularity within particular types of stimulus- or response-based information, which allows the independent learning of predictable series of events within individual dimensions. In addition, a multidimensional module allows sequence learning across types of information. Associations within the multidimensional system involve integration of stimulus properties, such as shape and spatial position, color and spatial position, and shape and auditory frequency. In addition, some studies have shown evidence of integration using conceptual stimuli (e.g., Cock and Meier, 2007; Meier and Cock, 2010), space, and temporal sequences (e.g., Shin and Ivry, 2002), and even a tone-counting task and SRT task (Rah et al., 2000; Hsiao and Reber, 2001). Therefore, due to the engagement of multiple sensory-specific modules, the secondary sequence can be integrated into the primary sequence.

Participants might be able to learn both the primary and secondary sequences and to integrate them into the overall representation of the task, but it is still not clear whether the integrated sequence leads to better enhancement of learning compared to only the primary sequence. Some studies have adopted a direct one-to-one association between the primary and secondary sequence and investigated whether the combined sequence leads to better learning compared to the primary sequence (e.g., Wright and Shea, 1991; Robertson and Pascual-Leone, 2001; Abrahamse et al., 2009, 2012). For example, Robertson and Pascual-Leone (2001) used three types of the implicit SRT task: the position, color, and combined tasks. In the position task, a target was presented at one of four horizontally aligned positions and four response buttons were pressed in accordance with the possible stimulus positions (i.e., a typical response-based learning). In the color task, a colored target was always presented in the center of the screen. Each of four colors was assigned to one of four response buttons (i.e., stimulus-based learning). In the combined task, spatially colored targets were used; the colored target was presented at one of four positions, and its color corresponded to the button position (i.e., both response- and stimulus-based learning). The results showed that the combined sequence led to greater effects of sequence learning than the other two sequences. These results suggest that sequence learning is enhanced when multiple sources of information are assigned to the same response (see also Robertson et al., 2001). However, in a later study, Abrahamse et al. (2012) adopted a similar experimental paradigm to that of Robertson and Pascual-Leone (2001) but they did not find supportive results (see also Abrahamse et al., 2009). They argued that the shorter performance time in the combined task in Robertson and Pascual-Leone (2001) was because the difficulty of the sequence learning among the tasks was not controlled, the *Z*-transformed scores were adopted instead of absolute differences in reaction time, and the number of participants was relatively small (i.e., four participants).

Given that a combined sequence does not enhance sequence learning, three possibilities arise. The first possibility is that the effects of stimulus-based learning are weaker than those of response-based learning. For example, the performance time for the colored sequence was slower than that for the position sequence (Abrahamse et al., 2012). According to the dual system model (Keele et al., 2003), the lack of benefits from a secondary sequence on the SRT performance might be because one of the systems was too slow to improve the performance (here, the color sequence). The second possibility is that the SRT task using a one-to-one association may lead to less engagement in the color sequence. Since the space-color association is fixed during performance, participants are likely to attend to a response-based sequence that can be processed faster than a stimulus-based sequence. More importantly, participants did not need to intentionally acquire the correct order of the button presses in the SRT task because some studies focused on implicit learning (e.g., Nissen and Bullemer, 1987). Therefore, the effects of the secondary sequence on the primary sequence might originally be small. A third possibility is that the integration of the secondary sequence into the primary sequence requires attentional or/and cognitive resources; the development of the multidimensional system may involve costs in the dual system model (Keele et al., 2003). Numerous studies have investigated whether the SRT learning is impaired if attentional resources are occupied by a secondary task (see Shanks, 2003, for a review). Given that selective attention is necessary for implicit learning (Jiménez and Méndez, 1999, 2001), a detrimental effect on sequence learning is observed. That said, Cock et al. (2002) suggested that selective attention is not necessary when attention for sequence learning is reduced in a dual-task condition, such as a symbol-counting task in addition to the SRT task (Shanks et al., 2005).

Together with these three possibilities, and given that learning of the color sequence was relatively slow to improve and/or integration of the color sequence into the spatial response sequence costs the resources, the combined sequence might lead to slow learning. That said, the time course of learning in the combined sequence and how the learning differs in the early and late learning phases in the combined and primary sequences are unclear. According to one theory of sequence learning (Hikosaka et al., 1999; Bapi et al., 2000, 2006), spatial and motor systems exist. In the spatial system, the spatial configurations of the button presses are learned (i.e., effector-independent learning), which mainly occurs in the early learning phase. In the motor system, the motor representations of the sequence are developed (i.e., effector-dependent learning), which mainly occurs in the late learning phase. The spatial and motor systems can work in parallel, but the time courses of development are different.

**FIGURE 1:**
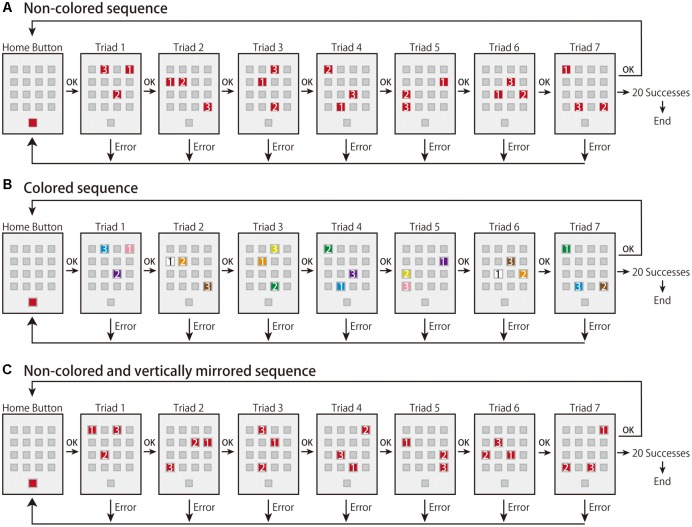
Experimental paradigms in the present study. **(A)** The experimental flow of the non-colored sequence. **(B)** The experimental flow of the colored sequence. The color of the buttons was either orange, yellow, green, cyan, violet, pink, white, or brown. Once the colors were assigned to buttons, the connection was kept during the experiment. **(C)** The experimental flow of the vertically mirrored sequence. Note that the numbers shown on the illuminated buttons were not presented during the experiments. **(A,C)** Adapted from Tanaka and Watanabe (2014a). Copyright 2014 by Elsevier.

In the present study, we aimed to investigate the effects of the sequence integrated in terms of space and color on the early and late learning phases (corresponding to effector-independent and effector-dependent learning, respectively) and how the effects differed in the integrated and primary sequences in each learning phase. To avoid low engagement in the task, we employed a visuomotor sequence-learning task in which participants need to decipher a predetermined correct order of a sequence using trial-and-error, which is known as the *m* × *n* task (e.g., Hikosaka et al., 1995, 1996, 2002; Watanabe et al., 2006, 2010; Tanaka and Watanabe, 2013, 2014a,b, 2016; **Figure 1**). In the task, participants are required to perform a sequence using trial-and-error. Sixteen placeholders (hereafter, called “buttons”) in a 4 × 4 matrix were drawn on a touch screen. In the 3 × 7 task, three buttons (i.e., a triad) turn on at the same time (*m*) and there are seven triads (*n*) in a sequence. As all triads have a predetermined correct order of buttons to be pressed, participants are required to learn the sequence using trial-and-error. In the present study, we prepared two types of sequence: non-colored and colored. In the non-colored sequence, the three buttons were illuminated in the same red color (i.e., only a spatial response sequence), while in the colored sequence, they were illuminated in different colors (i.e., a combined sequence of spatial responses and color stimuli). First, all participants performed the non-colored sequence as the baseline task, and they were then randomly assigned to two groups: a non-colored group or a colored sequence group. Then, they performed the non-colored or colored sequence as the learning task. Note that those in the colored sequence group were instructed to attend to both the color and spatial response sequences as much as possible in order to not let them focus on only one sequence. Finally, in order to investigate whether participants can use their obtained knowledge or motor representations in the learning task, all participants performed a non-colored sequence as a transfer task, in which the spatial configurations of the buttons were vertically mirrored from the learning task.

In line with evidence of the learning of multiple sources (e.g., Keele et al., 2003), we hypothesized that participants in the colored sequence group would combine the spatial response and color sequences in the early learning phase; that is, the colored sequence would lead to earlier acquisition of the correct button presses (i.e., fewer error trials) than the non-colored sequence. Otherwise, in an explicit learning situation requiring more engagement than the SRT task, the participants would not take advantage of the color stimulus sequence even if they tried to use it as much as possible. After the acquisition of the correct button presses, two possibilities could arise for the performance time. If the colored sequence leads to a shorter performance time than the non-colored sequence, this would indicate that multiple sources of information enhance effector-dependent learning in the explicit learning situation. This would suggest that once a multidimensional system is established in the dual system model (Keele et al., 2003), the multidimensional system enhances the overall processes (i.e., each unidimensional module). Alternatively, the time might be slower in the colored sequence than in the non-colored sequence. Together with the parallel learning of the effector-independent and effector-dependent representations (Hikosaka et al., 1999), this would suggest that combining the sequences requires additional attentional and/or cognitive resources in the effector-independent learning compared to the performance of only the spatial response sequence and would result in a delay in the development of the effector-dependent learning. In addition, in the transfer task (in which all participants perform the non-colored sequence), if the performance levels of the non-colored and colored sequence groups are significantly different, this would suggest that the colored sequence in the learning task affects the subsequent performance of the non-colored and vertically mirrored sequence.

## Materials and Methods

### Participants

Thirty-nine paid volunteers (24 men, 15 women; mean age = 20.94 years, standard deviation = 1.66; 38 right-handed according to self-reports) participated in the present study. All participants had normal motor functions, and were naïve to the purpose of this study. The experiment was approved by the institutional review board of The University of Tokyo. These experiments were conducted in accordance with the ethical standards in the 1964 Declaration of Helsinki. All of the participants provided informed consent prior to the study.

### Baseline Task

In the explicit *m* × *n* task (**Figure 1A**), all button stimuli were presented on a 19-inch touch panel monitor (ET1928L; Elo Touch Solutions). Sixteen placeholders (i.e., buttons) were shown in 4 × 4 matrix in the center of the monitor, and another button, called the “home button,” was displayed in the bottom of the monitor. Each button was a 3.5 cm square and the space between the buttons was 1.5 cm.

Before the start of each trial, the 16 buttons turned dark gray and the home button turned red against the light gray background. All participants used the index fingers of their dominant hand to press the buttons. Immediately after the home button was pressed, it turned dark gray and three out of the 16 buttons (i.e., a “triad”) turned red simultaneously. The triad had a predetermined correct order that needed to be revealed by trial-and-error. A sequence was composed of seven triads. The red buttons turned dark gray one by one when the button was correctly pressed. After the three buttons were correctly pressed, the next triad turned red. When a button was wrongly pressed, all buttons briefly turned red with a beep sound, and the next trial started from the beginning of the sequence (i.e., the home button). A trial was judged as a success only when the seven triads of the sequence were consecutively executed without any errors. A trial was judged as an error when a button was wrongly pressed in any of the triads. The task ended when the participant performed the same sequence successfully for 20 trials. During the task, once participants pressed the home button to start a trial, they were required to perform the sequence as accurately and quickly as possible, and they were allowed a brief break while the home button turned red (i.e., before the start of a trial).

In the present study, four types of sequence were randomly created, one of which was randomly assigned to participants as the baseline task. One of the remaining three sequences was randomly selected and used for the following learning task. The four sequences were created to avoid sharing the same dyads, triads, and repetitions; therefore, the unexpected transfer of the baseline sequence was unlikely to happen in the following learning and transfer tasks. Moreover, in the sequence creation, we tried to reduce the saliency of the repetitions in the sequence structure. The present sequence had 21 button presses (3 × 7 sequence) although the available buttons were 16 (4 × 4). Therefore, five buttons were inevitably repeated twice in the sequence, but no buttons were repeated more than three times.

### Learning Task

For the learning task, we prepared two types of sequence: non-colored and colored. We assigned the participants to either non-colored (*n* = 19) or colored sequence group (*n* = 20). In the non-colored sequence (**Figure 1A**), the task procedure was identical to that in the baseline task. In contrast, in the colored sequence (**Figure 1B**), the task procedure was also identical to that in the baseline task except that different colored buttons were turned on. In the colored sequence, we prepared eight types of color: orange, yellow, green, cyan, violet, pink, white, and brown. We randomly assigned the eight colors to the 16 buttons for each participant; two buttons shared the same color and the mappings between color and button were consistent throughout the learning task. Note that the probability of the colored sequence having at least one same color combination in seven triads was approximately 79%. While a triad turned on each assigned color, the other buttons turned gray. When the button press was correct, the buttons turned dark gray one by one. Before the task commenced, we simultaneously presented the eight colored squares in the screen and confirmed that the all participants could discriminate between the eight colors. We also instructed the participants in the colored sequence group to use the color sequence as much as possible.

### Transfer Task

In the transfer task, we used a non-colored sequence in which button configurations were vertically mirrored from the learning task (**Figure 1C**; Tanaka and Watanabe, 2014a). Tanaka and Watanabe (2014a) showed that the vertically mirrored configuration effectively led to transfer of learning compared to horizontally mirrored and rotated configurations. The reason we adopted the vertically mirrored sequence was to see whether the weaker learning deteriorated performance in transfer given that weaker learning occurred in the colored sequence; the easiest sequence rule was adopted. In addition, we used only the non-colored sequence and not the colored sequence in the present study because we focused on whether sequence learning using the multiple cues led to a better or worse transfer than when a single source of information of sequence was used (i.e., only spatial response sequence).

The participants in both colored and non-colored sequence groups performed the non-colored transfer sequence. Before the start of the transfer task, the participants were informed that the transfer sequence was vertically mirrored from the learning sequence. The other procedures were identical to those of the baseline and learning tasks. The participants performed the baseline, learning, and transfer tasks. Note that in the baseline and transfer tasks, both colored sequence and non-colored sequence groups performed the non-colored sequence, whereas in the learning task, the colored and non-colored sequence groups performed the colored and non-colored sequences, respectively.

### Data Analysis

We independently measured error and performance time, as in previous studies (e.g., Watanabe et al., 2006, 2010; Tanaka and Watanabe, 2013, 2014a,b). Error refers to the number of error trials before completing one trial. Performance time refers to the performance time only in successful trials (i.e., the time from when the home button was pressed to when the third button of the seventh triad was pressed). For both error and performance time, we divided the whole performance into five trial sections based on the number of successful trials (i.e., until the fourth, from after the fourth to the eighth, from after the eighth to the 12th, from after the 12th to the 16th, from after the 16th to the 20th successful trial), counted the total number of error trials, and calculated the mean performance time within each trial section. For example, the errors in the second trial section indicated the number of committed errors from after the fourth successful trial to the eighth successful trial, and the performance time in that section indicated the mean performance time from the fifth to the eighth successful trial. We mainly conducted mixed two-way analysis of variance (ANOVA) with the five trial sections as within-subject factors and the two types of groups (colored and non-colored sequence groups) as between-subject factors. In *post hoc* tests, Shaffer’s method was used where appropriate. Effect sizes ($\eta_{p}^{2}$) were calculated for all of the ANOVAs.

In addition, we measured the total number of button presses and total working time. The total number of button presses indicated the cumulative number of button presses in both successful and error trials, and did not include the presses of the home button. The total working time indicated the cumulative performance time in both successful and error trials, but did not include the time while the home button was red. We also calculated the mean button press time by dividing the total working time by the total number of button presses in the task. In the data analysis, we separated these two measures into two parts: the performance until the first successful trial and those from after the first successful trial to the 20th successful trial. Finally, we compared each measurement between the colored and non-colored sequence group using two-sample *t*-tests. Cohen’s *d* was used for the two-sample *t*-tests.

## Results

One participant in the colored sequence group was excluded from further analysis because the mean performance time in the baseline task was slower by two standard deviations from the colored sequence group’s average, resulting in 18 and 20 participants in the colored and non-colored sequence groups, respectively.

### Baseline Task

**FIGURE 2:**
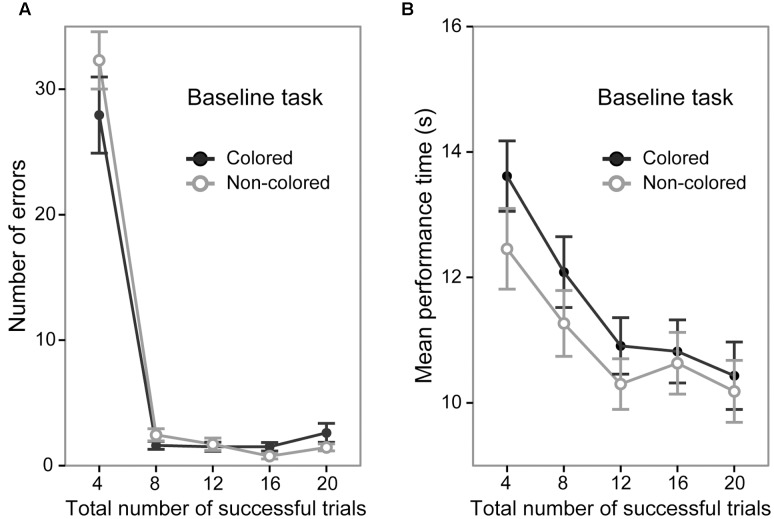
Performance of the colored and non-colored sequence groups in the baseline task. Error bars show the standard errors of the mean. **(A)** Average number of errors before the successful completion of each trial. **(B)** Average performance time for successful trials.

A 5 (trial section) × 2 (sequence group) ANOVA of the number of error trials revealed a significant main effect of trial section [*F*(4,144) = 223.99, *p* < 0.0001, $\eta_{p}^{2}$ = 0.86; **Figure 2A**] and *post hoc* tests showed that the number of error trials in the first section (mean = 30.12 times) was significantly larger than that in the other sections (mean = 2.03, 1.60, 1.12, and 2.03 times, in the second, third, fourth, and fifth trial sections, respectively, *p* < 0.0001). The ANOVA did not show a significant main effect of sequence group [*F*(1,36) = 0.60, *p* = 0.44]. The interaction between trial sections and sequence type was not significant [*F*(4,144) = 1.66, *p* = 0.16]. The mean number of error trials in the fifth trial section was 2.61 (95% *CI* [1.42 3.79]) and 1.45 (95% *CI* [0.72 2.17]) in the colored sequence and non-colored sequence group, respectively.

A 5 (trial section) × 2 (sequence group) ANOVA of the mean performance time in the successful trials showed a significant main effect of trial section [*F*(4,144) = 34.05, *p* < 0.0001, $\eta_{p}^{2}$ = 0.48; **Figure 2B**]. The results of *post hoc* tests indicated that the performance time gradually became faster (first > second > third = fourth = fifth, *p* < 0.01; mean = 13.03, 11.67, 10.60, 10.72, and 10.30 s in the first, second, third, fourth, and fifth sections, respectively). The ANOVA did not show a significant main effect of sequence group [*F*(1,36) = 0.85, *p* = 0.36]. The interaction between trial section and sequence group was not significant [*F*(4,144) = 1.12, *p* = 0.34]. The mean performance time in the fifth trial section was 10.43 s (95% *CI* [9.92 10.94]) and 10.18 s (95% *CI* [9.79 10.56]) in the colored sequence and non-colored sequence group, respectively. Taken together with the results regarding the number of errors, the both groups reached similar performance level at the end of the baseline task.

Regarding the performance until the first successful trial, we compared the total number of button presses, total working time, and mean button press time (total working time until the first successful trial/total number of button presses until the first successful trial) of the two sequence groups. Two-sample *t*-tests did not show significant differences between the non-colored and colored sequence group for the total number of button presses [*t*(36) = 0.63, *p* = 0.53, *d* = 0.20; colored sequence group, mean = 247 times; non-colored sequence group, mean = 268 times], total working time [*t*(36) = 0.075, *p* = 0.94, *d* = 0.024; colored sequence group, mean = 187 s; non-colored sequence group, mean = 189 s], and mean button press time [*t*(36) = 0.41, *p* = 0.68, *d* = 0.13; colored sequence group, mean = 731 ms; non-colored sequence group, mean = 716 ms].

Similarly, for the performance from after the first successful trial to the 20th successful trial, two-sample *t*-tests did not show significant differences between the non-colored and colored sequence group regarding the total number of button presses [*t*(36) = 0.37, *p* = 0.70, *d* = 0.12; colored sequence group, mean = 483 times; non-colored sequence group, mean = 490 times], total working time [*t*(36) = 0.50, *p* = 0.61, *d* = 0.16; colored sequence group, mean = 261 s; non-colored sequence group, mean = 272 s], and mean button press time [*t*(36) = 0.13, *p* = 0.89, *d* = 0.044; colored sequence group, mean = 544 ms; non-colored sequence group, mean = 549 ms].

The results of the baseline task confirmed that the performance in the baseline task did not significantly differ between the colored and non-colored sequence groups. The relatively rapid improvement of accuracy (i.e., the number of errors) and slow improvement of speed (i.e., mean performance time) reflects a different time course of acquisition of accuracy and speed. Identical results have been reported in previous works (e.g., Hikosaka et al., 1995, 1996, 1999; Sakai et al., 1998; Watanabe et al., 2006, 2010; Tanaka and Watanabe, 2013, 2014a,b, 2016).

### Learning Task

**FIGURE 3:**
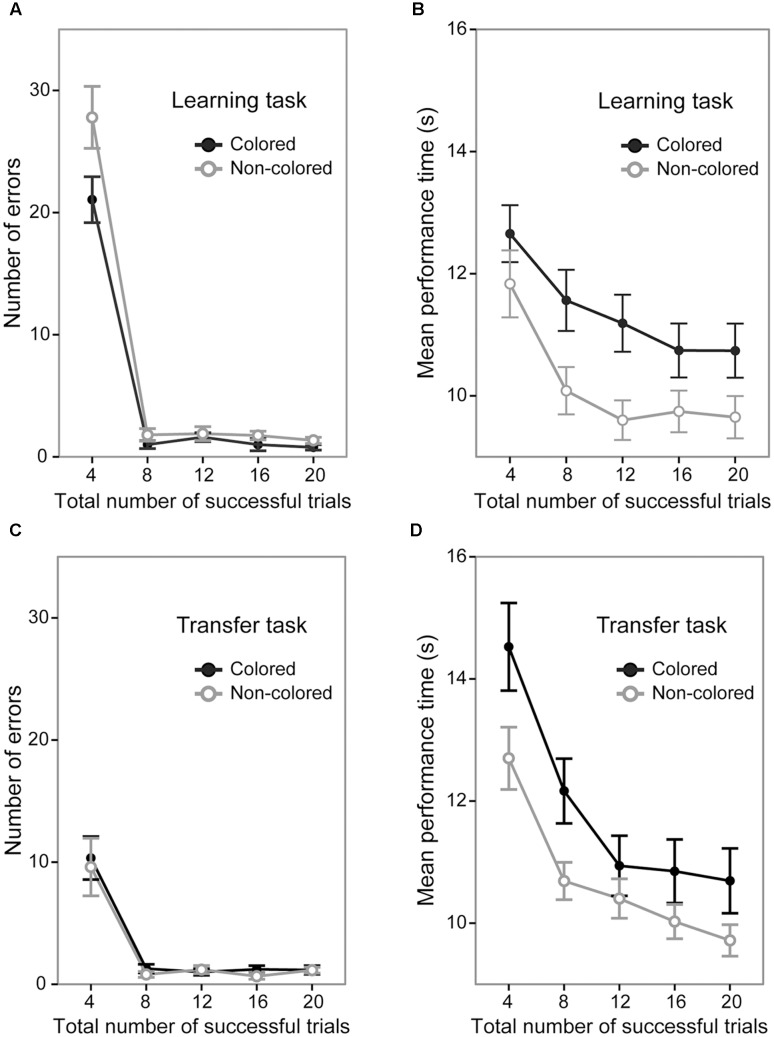
Performance of the colored and non-colored sequence groups in the learning and transfer tasks. Error bars show the standard errors of the mean. Note that in the transfer task, both the colored and non-colored sequence group performed the non-colored sequence. **(A)** Average number of errors before the successful completion of each trial in the learning task. **(B)** Average performance time for successful trials in the learning task. **(C)** Average number of errors before the successful completion of each trial in the transfer task. **(D)** Average performance time for successful trials in the transfer task.

A 5 (trial section) × 2 (sequence group) ANOVA of the number of error trials revealed significant main effects of trial section [*F*(4,144) = 191.37, *p* < 0.0001, $\eta_{p}^{2}$ = 0.84; **Figure 3A**] and sequence group [*F*(1,36) = 5.92, *p* < 0.001, $\eta_{p}^{2}$ = 0.14]. The interaction between trial sections and sequence type was also significant [*F*(4,144) = 3.41, *p* < 0.05, $\eta_{p}^{2}$ = 0.08]. The signifiant interaction showed that in the first trial section, the number of errors was significantly larger in the non-colored sequence group (mean = 27.80 times) than in the colored sequence group [mean = 21.05 times; *F*(1,36) = 4.39, *p* < 0.05, $\eta_{p}^{2}$ = 0.10] while it was not significantly different in the other sections [*Fs*(1,36) < 2.81, *ps* > 0.10]. This result indicates that by the end of the first trial section, the participants in the colored sequence group acquired the correct button presses of the sequence earlier than those in the non-colored sequence group, but after the first trial section, errors rarely happened in both the colored and non-colored sequence groups.

A 5 (trial section) × 2 (sequence group) ANOVA of the mean performance time in the successful trials showed significant main effects of trial section [*F*(4,144) = 34.87, *p* < 0.0001, $\eta_{p}^{2}$ = 0.49; **Figure 3B**] and sequence group [*F*(1,36) = 4.71, *p* < 0.05, $\eta_{p}^{2}$ = 0.11]. The interaction between trial section and sequence type was not significant [*F*(4,144) = 1.26, *p* = 0.28]. This result indicates that the mean performance time in the non-colored sequence group (mean = 10.18 s) was generally shorter than in the colored sequence group (mean = 11.37 s).

Regarding the performance until the first successful trial between the colored and non-colored sequence groups, two-sample *t*-tests showed a significant difference of total number of button presses [*t*(36) = 2.15, *p* < 0.05, *d* = 0.70; colored sequence group, mean = 185 times; non-colored sequence group, mean = 248 times] and a marginally significant difference of mean button press time [*t*(36) = 1.91, *p* = 0.063, *d* = 0.62; colored sequence group, mean = 734 ms; non-colored sequence group, mean = 668 ms], but did not show a significant difference of total working time [*t*(36) = 1.46, *p* = 0.15, *d* = 0.47; colored sequence group, mean = 135 s; non-colored sequence group, mean = 163 s]. The larger number of button presses in the non-colored sequence group than in the colored sequence group reflected the larger number of error trials in the non-colored sequence group.

As for the performance from after the first successful trial to the 20th successful trial of the colored and non-colored sequence groups, two-sample *t*-tests did not show significant differences of total number of button presses [*t*(36) = 0.37, *p* = 0.70, *d* = 0.12; colored sequence group, mean = 467 times; non-colored sequence group, mean = 474 times] and total working time [*t*(36) = 1.30, *p* = 0.19, *d* = 0.42; colored sequence group, mean = 253 s; non-colored sequence group, mean = 233 s], but did show a marginally significant difference of mean button press time [*t*(36) = 1.94, *p* = 0.060, *d* = 0.63; colored sequence group, mean = 543 ms; non-colored sequence group, mean = 491 ms]. The results of the total number of button presses and working time indicate that after the first successful trial, the performance of the colored and non-colored sequence groups was not significantly different. However, the mean button press time tended to be shorter in the non-colored sequence group than in the colored sequence group, which reflected shorter performance time in successful trials by the non-colored sequence group than by the colored sequence group.

Taken together, the results of the learning task demonstrated that by the first trial section, the colored sequence led to earlier acquisition of the correct button presses of the sequence than the non-colored sequence did, but afterward, there were no significant differences regarding the number of errors. We also found that the mean performance time in the colored sequence group was generally slower than in the non-colored sequence group.

### Transfer Task

A 5 (trial section) × 2 (sequence group) ANOVA of the number of error trials revealed a significant main effect of trial section [*F*(4,144) = 32.98, *p* < 0.0001, $\eta_{p}^{2}$ = 0.47; **Figure 3C**], but did not show a significant main effect of sequence group [*F*(1,36) = 0.26, *p* = 0.61]. The interaction between trial sections and sequence type was not significant [*F*(4,144) = 0.08, *p* = 0.98]. This result indicates that the number of errors was not significantly different between the colored and non-colored sequence groups (15.00 times vs. 13.4 times). Note that although we found a significant difference of the number of errors between the groups in the learning session, we do not think that their performances in the transfer session were improved or deteriorated from the learning session. At the end of the learning session, the colored sequence group acquired the correct order of spatial button presses in addition to the colored sequence and the non-colored sequence group acquired only the correct order of spatial button presses. In the transfer session, we investigated how the sequential representations obtained in the learning session affected performances in the transfer session. In addition, at the beginning of the learning session, the both colored and non-colored sequence groups did not know the correct order of the sequence. In contrast, at the beginning of the transfer session, they knew the transfer rule. Therefore, in the present study, we did not focus on the change ratio from the learning session to the transfer session.

A 5 (trial section) × 2 (sequence group) ANOVA of the mean performance time in the successful trials showed significant main effects of trial section [*F*(4,144) = 55.37, *p* < 0.0001, $\eta_{p}^{2}$ = 0.60; **Figure 3D**] and sequence group [*F*(1,36) = 4.11, *p* < 0.05, $\eta_{p}^{2}$ = 0.10]. The interaction between trial section and sequence type was not significant [*F*(4,144) = 1.92, *p* = 0.11]. This result indicates that the mean performance time was shorter in the non-colored sequence group (mean = 10.70 s) than in the colored sequence group (mean = 11.83 s). The results of the transfer task showed a slower performance time in the colored sequence group, but did not show a significant difference of the number of errors between the sequence groups.

For the performance until the first successful trial of the colored and non-colored sequence groups, two-sample *t*-tests did not show significant differences of total number of button presses [*t*(36) = 0.39, *p* = 0.69, *d* = 0.12; colored sequence group, mean = 80 times; non-colored sequence group, mean = 91 times], total working time [*t*(36) = 0.039, *p* = 0.96, *d* = 0.012; colored sequence group, mean = 74 s; non-colored sequence group, mean = 73 s], and mean button press time [*t*(36) = 0.67, *p* = 0.50, *d* = 0.22; colored sequence group, mean = 947 ms; non-colored sequence group, mean = 891 ms]. These results indicate that until the first successful trial, the performance was not significantly different.

Regarding the performance from after the first successful trial to the 20th successful trial of the colored and non-colored sequence groups, two-sample *t*-tests did not show significant differences of total number of button presses [*t*(36) = 0.075, *p* = 0.93, *d* = 0.024; colored sequence group, mean = 454 times; non-colored sequence group, mean = 453 times] or total working time [*t*(36) = 1.44, *p* = 0.15, *d* = 0.46; colored sequence group, mean = 254 s; non-colored sequence group, mean = 233 s], but did show a marginally significant difference of mean button press time [*t*(36) = 1.79, *p* = 0.080, *d* = 0.58; colored sequence group, mean = 561 ms; non-colored sequence group, mean = 513 ms]. The tendency of the shorter mean time of button presses in the non-colored sequence than in the colored sequence reflected shorter performance time in successful trials in the non-colored sequence group than in the colored sequence group.

In sum, the results of the transfer task showed that the total number of errors was not significantly different between the colored and non-colored sequence groups. However, the mean performance time in the non-colored sequence group was shorter than that in the colored sequence group, as in the learning task^1^.

## Discussion

In the present study, we examined the effects of the combined sequence on effector-dependent and effector-independent learning in an explicit learning situation. The present results showed that the participants who performed the colored sequence acquired the correct button presses of the sequence earlier, but showed a slower mean performance time than those who performed the non-colored sequence. Moreover, the slower performance time in the colored sequence group remained in a subsequent transfer task in which the spatial configurations of the buttons were vertically mirrored from the learning task. These results indicate that the colored sequence group could develop effector-independent representations earlier, but were not able to effectively enhance their effector-dependent representations in the learning session. Thus, the undeveloped effector-dependent representations in the learning session in the colored sequence group directly led to a long performance time in the transfer sequence.

### Learning Task

The present results and the dual system model (i.e., Keele et al., 2003) together indicate that the colored sequence group could integrate the color stimulus sequence into the spatial sequence in the early learning phase (i.e., multidimensional system) but that there were attentional and cognitive costs for the integration. That is, they deliberately attended to both the spatial and color sequences, which likely produced a delay in the development of the effector-dependent representations compared with the non-colored sequence group. Therefore, at the completion of the first successful trial, the colored sequence group could not develop the effector-dependent representations more than the non-colored sequence group; this sluggishness lasted even to the end of the learning session. The present study is the first to report the time course of the effects of the combined sequence on sequence learning.

Several studies have demonstrated that a secondary sequence can be integrated into a primary sequence through practice regardless of the stimulus type (e.g., shape, color, or tone; Cock and Meier, 2013). In Cock and Meier’s (2013) experiment, participants were instructed to attend to only one stimulus and to ignore another stimulus. It was found that, even if they ignored the response-irrelevant sequence, they implicitly integrated the response-irrelevant sequence into the response-relevant sequence if both of the sequences shared the same length of elements and had an overall representation of the task. In the present study, we adopted a one-to-one association in the colored sequence; participants were explicitly able to learn the relationships between successive stimuli (S-S), successive responses (R-R), successive associations of stimuli and response (S-R), and responses and the next stimulus (R-S). Therefore, the redundancies in the relationships led to a smaller number of error trials and button presses in the colored sequence group than in the non-colored sequence group in the early learning phase, indicating the integration of the sequences. As few studies have adopted the error rate as a variable because errors rarely happen in the SRT task, the present study is the first to indicate that the combined sequence mainly helps the learning of effector-independent representations in the early learning phase (i.e., a reduction of error trials).

The non-enhancement of the mean performance time in the colored sequence group could be explained by the cost of integrating the spatial response and color sequences and the role of the multidimensional system. In the colored sequence group, the participants were asked to attend to both sequences for integration, while those in the non-colored sequence group attended to only the spatial sequence. Shanks et al. (2005) suggested that reaction times for a response task were shorter in a single-task condition than in a dual-task condition, indicating that if attention to the response task was attenuated, the learning of the motor sequence may not be enhanced. This was demonstrated by the tendency of the colored sequence group to have a slower button press time. That is, the deliberate attention to the color and space response sequences and the integration of both sequences required attentional and cognitive resources, resulting in the occupation of the effector-independent learning. As such, this probably led to the slower development of the effector-dependent learning in the early learning phase in the colored sequence group than in the non-colored sequence group.

More importantly, we did not find a significant interaction between the sequence group and trial section regarding mean performance time. This indicates that the slower performance in the colored sequence group lasted even to the end of the learning session. In other words, even after the acquisition of the effector-independent representations, the improvement ratio of the effector-dependent representations did not differ between the colored and non-colored sequence groups; that is, the multidimensional system in the colored sequence group did not contribute to additional enhancement of the effector-dependent representations. This finding is in line with previous works (e.g., Abrahamse et al., 2009, 2012). Abrahamse et al. (2012) did not find supportive results for the enhancement when using colors and spatial sequences. Furthermore, Abrahamse et al. (2009) used tactile and spatial sequences, but they did not find a significant enhancement. Taken together with the previous studies, participants could learn both primary and secondary sequences as long as both sequences were correlated, regardless of the stimulus properties (e.g., Shin and Ivry, 2002; Cock and Meier, 2007; Meier and Cock, 2010), and they could eventually combine them into an overall representation through the multidimensional system. However, even after the integration, the multidimensional system did not contribute to the improvement of the effector-dependent representations.

### Transfer Task

In Cock and Meier (2013), participants attended to a specific color asterisk (response-relevant sequence) and ignored another asterisk (response-irrelevant sequence), which were simultaneously presented at different locations. They found that performance times were disrupted when the response-irrelevant sequence became a random sequence after practice, but the response-relevant sequence did not change. This indicates the existence of three working systems; two that are each a unidimensional system for the response-relevant and irrelevant sequences, and another that is a multidimensional system that combines and monitors the two systems. Thus, when the response-irrelevant sequence is abruptly changed, the inconsistency between the response-relevant and response-irrelevant sequences interferes with the multidimensional system, which also results in interference with the unidimensional system for the response-relevant sequence, leading to disrupted performance times in the response-relevant sequence.

In the transfer task, all participants performed the non-colored sequence that was vertically mirrored from the learning task. The non-significant difference between the colored sequence group and non-colored sequence group suggests two possibilities. One is that the colored sequence group was not influenced by the multidimensional system in the transfer session. Similarly to Cock and Meier (2013), we could assume that the colored sequence group established the three working systems at the end of the learning session. Therefore, this suggests that when the colored sequence group performs the non-colored sequence, the unidimensional system for the color sequence and the multidimensional system do not work and do not interfere with the unidimensional system for the spatial response sequence. In other words, following Cock and Meier (2013), if a randomly generated colored sequence was included in the transfer session, the unidimensional system for the spatial response sequence would be interfered with. Another possibility is that as the number of errors was lower in the transfer task than in the learning task (i.e., it was easier), the non-significant difference may also reflect a floor effect. These issues need to be investigated in future studies.

The mean performance time in the successful trials was still significantly slower in the colored sequence group than in the non-colored sequence group. Two possibilities arise that might account for this finding. One possibility is that there are contextual dependencies in sequence learning. For example, Wright and Shea (1991) found that reaction times became slower when the position, color, sound, and shape of the stimuli in the sequence changed but the sequence was identical compared to when the stimulus properties did not change, reflecting contextual dependencies of sequence learning. The participants in the colored sequence group could not observe color information in the transfer task; thus, contextual dependencies regarding the color have might occurred. That said, the present experiment used only color while Wright and Shea (1991) adopted position, color, sound, and shape, which may have resulted in relatively weaker effects of contextual dependencies. The second possibility is that the slower performance time in the colored sequence group in the transfer task may reflect the slower performance time at the end of the learning task in the colored sequence group compared to in the non-colored sequence group. At the end of the learning task, the participants in the colored sequence group did not reach the same level of speed as those in the non-colored sequence group. Thus, the slower motor performance might remain even in the transfer task. As such, the colored sequence group did not show any superiority to the non-colored sequence group in the transfer task.

In the present transfer task, we used only the non-colored sequence and not the colored sequence because we focused on whether sequence learning with multiple cues led to better or worse transfer than when a single cue sequence was used (i.e., only the spatial response sequence). The use of two types of colored sequence in the transfer task could be considered. One type would involve the locations of the color stimuli and spatial buttons in the sequence being vertically mirrored in the transfer task, resulting in an identical colored sequence to that in the learning sequence. Hence, the participants in the colored sequence group would follow only the colored sequence and would not need to transfer the spatial sequence. In this case, we might be able to examine how well the participants could use the colored sequence by comparing them to those who performed the colored sequence in the learning task and the non-colored sequence in the transfer task. Since the results of the learning task in the colored sequence group showed earlier acquisition of the spatial representations of the sequence, it could be presumed that better transfer would occur regarding the number of errors if the locations of the color stimuli in the sequence were vertically mirrored to those in the current experimental groups. The other type would involve the locations of the color stimuli in the sequence not being vertically mirrored in the transfer task (i.e., only the spatial button configuration being vertically mirrored), resulting in a different colored sequence from that in the learning task. Here, the participants would be required to rely on only the spatial sequence and to ignore or learn the unlearned colored sequence. By comparing them to those who performed the colored sequence in the learning task and the non-colored sequence in the transfer task, we could examine if the different colored sequence in the transfer task interferes with performance in the transfer task in terms of the number of errors and the performance time.

## Author Contributions

Conceived and designed the experiments: KT and KW. Performed the experiments: KT. Analyzed the data: KT. Contributed reagents/materials/analysis tools: KT and KW. Wrote the paper: KT and KW.

## Conflict of Interest Statement

The authors declare that the research was conducted in the absence of any commercial or financial relationships that could be construed as a potential conflict of interest.

## Footnotes

1 For further verifications of the present results, we calculated a speed index (%): (P^learningortransfer^ – P^baseline5th^) / P^baseline5th^. We subtracted mean performance time in the fifth trial section in the baseline session from performance time in the learning or transfer session and divided the value by the mean performance time in the fifth trial section in the baseline session. Negative value indicates that the performance time in the learning or transfer session was shorter than that in the baseline session and vice versa. We excluded three participants because their speed index in the learning or transfer session was larger than mean ± 2SD in each group, resulting in 18 participants in each group. A 2 × 5 ANOVA regarding speed index in the learning session showed a significant main effect of group [*F*(1,34) = 5.71, *p* < 0.05, $\eta_{p}^{2}$ = 0.14], but did not show any significant interaction [*F*(4,136) = 1.39, *p* = 0.23]. This indicates that the speed in the learning session was faster in the non-colored sequence group than in the colored sequence group. Next, a 2 × 5 ANOVA regarding speed index in the transfer session showed a tendency of a significant main effect of group [*F*(1,34) = 3.94, *p* = 0.055], but did not show any significant interaction [*F*(4,136) = 1.65, *p* = 0.16]. This indicates that the speed in the transfer session tended to be faster in the non-colored sequence group than in the colored sequence group. The unexpected small effect was likely due to the relatively large variance in the speed index. The range of speed index in the transfer session was from -14.73 to 42.23% (*SD* = 15.75) and from -29.73 to 26.05 % (*SD* = 14.43) in the colored and non-colored sequence groups while the range of mean performance in the transfer session was 8.35 to 15.68 s (*SD* = 2.07) and 8.28 to 13.55 s (*SD* = 1.30). This may indicate that performances in the transfer session have relatively large individual differences; some might be able to easily transfer their obtained knowledge in the learning session to the vertically mirrored sequence, but some might not. If we would adopt the individualized index not a raw performance time in the present experimental paradigm, we may need to confirm the present findings with a larger number of participants to cover the larger variance. Further studies need to be performed to investigate this issue.
